# Challenges of Diabetes Self-Management in Adults Affected by Food Insecurity in a Large Urban Centre of Ontario, Canada

**DOI:** 10.1155/2015/903468

**Published:** 2015-10-20

**Authors:** Justine Chan, Margaret DeMelo, Jacqui Gingras, Enza Gucciardi

**Affiliations:** ^1^Ryerson University, 350 Victoria Street, Toronto, ON, Canada M5B 2K3; ^2^University Health Network, 399 Bathurst Street, Toronto, ON, Canada M5T 2S8

## Abstract

*Objective.* To explore how food insecurity affects individuals' ability to manage their diabetes, as narrated by participants living in a large, culturally diverse urban centre. *Design.* Qualitative study comprising of in-depth interviews, using a semistructured interview guide. *Setting.* Participants were recruited from the local community, three community health centres, and a community-based diabetes education centre servicing a low-income population in Toronto, Ontario, Canada. *Participants.* Twenty-one English-speaking adults with a diagnosis of diabetes and having experienced food insecurity in the past year (based on three screening questions). *Method.* Using six phases of analysis, we used qualitative, deductive thematic analysis to transcribe, code, and analyze participant interviews. *Main Findings.* Three themes emerged from our analysis of participants' experiences of living with food insecurity and diabetes: (1) barriers to accessing and preparing food, (2) social isolation, and (3) enhancing agency and resilience. *Conclusion.* Food insecurity appears to negatively impact diabetes self-management. Healthcare professionals need to be cognizant of resources, skills, and supports appropriate for people with diabetes affected by food insecurity. Study findings suggest foci for enhancing diabetes self-management support.

## 1. Introduction

By 2020, the cost to treat diabetes mellitus, its complications, and its associated loss of productivity and life will exceed CAN$19 billion a year [1]. Compelling evidence supports the benefits of intensive glycemic, lipid, and blood pressure control in the prevention and management of diabetes complications [2]. However, diabetes self-management is a challenge for many people as it involves learning and adopting self-care and self-monitoring practices [3]. For instance, medical nutrition therapy, a cornerstone in diabetes management, is one of the most challenging aspects of management [4, 5], encompassing not only healthy eating, but also insulin dose adjustment to carbohydrate consumed, as well as prevention and treatment of hypoglycaemia.

Household food insecurity is significantly more common among Canadians with diabetes (9.3%) compared to Canadians without diabetes (6.8%) [6], which is similar to that of other countries [7, 8]. Food security exists “when all people, at all times, have physical and economic access to sufficient, safe, and nutritious food to meet their dietary needs and food preferences for an active and healthy life” [9]. Notably rooted in poverty, food insecurity for people with diabetes poses additional challenges, principally, the lack of adequate and appropriate food and the effect on diabetes management [10]. Studies of adults who are food insecure show that they are more likely to have poorer health, inferior social support, and more comorbid conditions (e.g., obesity, high blood pressure, heart disease, and allergies) [11] as well as greater psychological distress and unhealthy behaviours, including smoking, physical inactivity, and low consumption of fruits and vegetables [6, 11]. Specifically, food insecure adults with diabetes are more likely to have poor glycemic control, long-term complications, and severe and frequent hypoglycaemia [12–16]. While it is clear that those that are living with diabetes and are food insecure experience more health problems and poorer overall health, knowledge on how they cope and manage with this health intersection and information on their lived experiences to date have been primarily absent in the literature. This type of knowledge can provide insight on how to better support and care for this population.

Using in-depth interviews with adults living with diabetes and food insecurity, our research paper explores these lived experiences and tries to understand how food insecurity affects people's ability to manage their diabetes. The semistructured interview guide was based on the Social Determinants of Health Framework, given the significant influence of material deprivation and financial constraints on diabetes management decisions [17]. The findings will be novel as there is very limited qualitative research in this area.

## 2. Materials and Methods

### 2.1. Qualitative Methodology

There is little contextual research on how and why individuals with diabetes coping with food insecurity are more likely to practice unhealthy behaviours and endure greater psychological distress. Using six phases of analysis, we chose qualitative, deductive thematic analysis, as it is flexible in its approach to extract themes from participants' accounts [18].

### 2.2. Participant Recruitment

With the help of diabetes educators and flyers promoting the study, we conducted one-on-one interviews with clients from the local community, three community health centres, and a community-based diabetes education centre serving a low-income population in the Greater Toronto Area, Ontario, Canada. Eligibility criteria included a diagnosis of type 1 or 2 diabetes, English speaking, and having experienced food insecurity in the past year. Three questions were adapted from the Household Food Security Survey Module (HFSSM) [19] to identify participants: (1) In the past year, were you ever not able to buy your basic foods, such as fruits and vegetables? (2) In the past year, were you ever not able to buy your favourite foods? (3) In the past year, did you ever have to eat less than you felt you should have because of a limited budget? Eligibility was based on at least one affirmative response to the above questions. Homeless individuals were excluded, as the complexity of psychosocial vulnerabilities and mental health issues would extend far beyond the scope of our research question. See Table 1 for our sample demographics. Participant recruitment ended when theme saturation was achieved [20].

Ethics approval for this research study was obtained from Ryerson University's Research Ethics Board in Ontario, Canada (REB 2008-294). Initial data collection for this study was completed in 2009-2010. The four-member research team are comprised of one academic expert in diabetes (Enza Gucciardi), one expert in qualitative research (Jacqui Gingras), and two practice experts in diabetes (Justine Chan, Margaret DeMelo).

### 2.3. Procedure for Data Analysis

Eligibility, demographic information, and verbal informed consent were confirmed by the lead author prior to the interview. In total, 21 individual face-to-face interviews were conducted, lasting 30 to 90 minutes.

The semistructured interview guide is as follows.

*Introduction*

 Can you remember when you were first diagnosed with diabetes? What was it like?
 How did you react to the news?

*Health Services*

 Think back to when you first learned about what you would need to do to manage your diabetes. How was that experience for you?
 What did the doctor/nurse/and so forth tell you about what you had to do to manage your diabetes? What was it like learning about what you needed to do in terms of food, physical activity, and medication? What challenges did you face in trying to follow the recommendations of your physician or the diabetes health care team?
 Can you tell me when you first found it difficult to buy or find food? Was this before or after you found out you had diabetes?

*Income and Social Status*

 How did you manage through the times when you found it hard to buy or find food? How has this affected you in the past year? If they have kids or living with family: When providing meals for other people in the household, what were your major concerns?

*Social Environments/Social Support Networks*

 Were you able to get the help/assistance/understanding/support you felt you needed during these times?
 What support was available to you at this time (formal support from health/social care providers, family, friends, church group, etc.)? Would you tell a bit me about these experiences? What supports really made a difference for you; what was most helpful to you? What more can they (heath care providers, government, etc.) do to better help you?
 How have concerns around food affected your daily life?

*Personal Health Practices and Coping Skills*

 Some of the things we've been talking about sound like they may have been very difficult/challenging for you. What have been the biggest challenges overall? What have been your “survival” strategies through all this? How confident are you now in your ability to manage your diabetes?

*Cool-Down/Wrap-Up*

 Looking back on your own experiences, what would have made this whole journey easier for you?
 If you could send a message to other people in your situation, what would it be?
 What about healthcare providers? What would you like them to know?
 Is there anything else about this experience that was important to you that we haven't talked about?
 The responses you have provided may lead to some more questions. If so, can we contact you for a follow-up interview?

Probes were used to encourage participants to elaborate on their experiences. Participants received CAN$30 honorarium at the end of their interviews. All interviews were digitally recorded and transcribed verbatim. Following data collection, we removed all participant identifiers to protect anonymity.

Our analysis process required six phases [21], beginning with the research team reviewing interview transcripts, while making notes for potential codes to return to at a later stage in analysis. The second phase involved developing preliminary codes, 50 in total, to capture as many potential themes as possible. We grouped transcript excerpts under these codes both manually and by NVivo data management software. Following phase two, we created clusters of codes and grouped them into ten major themes using the repetition technique where recurring topics generated the most relevant ideas [21]. In the third phase, we used thematic networks [22] to systematically present the study findings by listing our themes from specific to broad, that is, basic, organizing, and global themes, respectively. In the fourth phase, we met frequently to further refine our themes and triangulate the data. The basic themes were then grouped under three overarching themes. In the fifth phase, we refined theme wording to capture the meaning of what was said. The sixth phase involved writing and revising the manuscript report, while the research team carefully considered the most meaningful extracts.

## 3. Results

**Figure 1:**
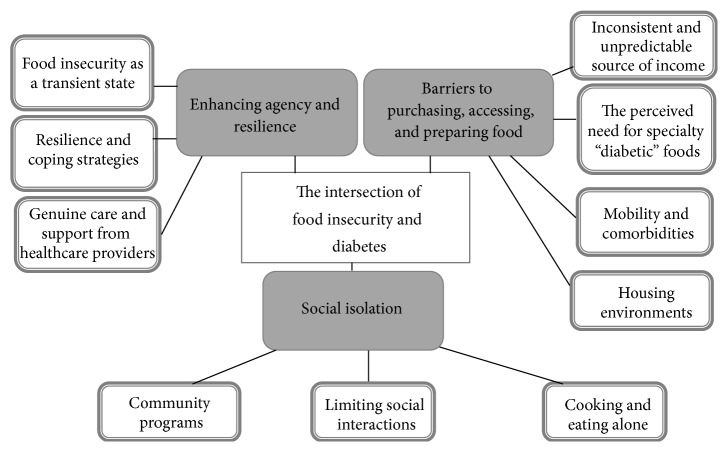
The intersection of food insecurity and diabetes.

Our data analysis produced three main themes that captured the experiences of people with diabetes who are food insecure: (1) barriers to preparing and accessing appropriate food, (2) social isolation, and (3) enhancing agency and resilience (see Figure 1).

### 3.1. Themes and Additional Supporting Quotes

**Theme 1 (barriers to preparing and accessing appropriate food).**

Most participants could not afford foods appropriate for their diabetes management. Always buying “healthy foods,” counting carbohydrates, or tracking serving amounts were unrealistic approaches to meal planning because food supplies were often erratic due to an inconsistent and unpredictable source of income. When grocery shopping, their goal was to buy whatever cost the least (see Interviewee 1). Participants also worried about not having enough food to eat and described the additional challenges brought on by a limited budget (see Interviewee 2). Meal planning was difficult and many depended on other resources, such as food banks and community kitchens. Inappropriate foods available at food banks (i.e., high in starch, salt, and sugar) were voiced (see Interviewees 4 and 5). Unfortunately, participants still struggled with access to these food sources (see Interviewee 3). For example, a single mother modified her work hours with the food bank's operating hours. Access to food was often better at the beginning of the month, resulting in more erratic blood sugars by month's end.

Housing environments presented barriers to food preparation. Many did not own a stove, resorting to microwaveable foods (see Interviewee 6). A lack of proper cooking facilities resulted in greater use of higher sodium foods such as processed and canned foods.

Misperception of the type of foods recommended for diabetes management was widely common. Participants discussed how specialty foods, such as those containing artificial sweeteners or labeled “diabetic,” were perceived as “better,” expensive, and, contrary to current nutrition recommendation, understood to be necessary for the management of diabetes (see Interviewees 7 and 9).

The barriers resulting from the intersection of diabetes and food insecurity were especially evident for those who had to cope with debilitating comorbidities. Retinopathy and neuropathy, in particular, greatly reduced their access and selection of both fresh and appropriate foods (see Interviewee 8). For example, many voiced the challenge of no longer being able to travel to food banks or grocery stores and had difficulty selecting and preparing food (see Interviewee 2).

Interviewee 1: I don't have a lot of money…so I'll buy junk food, instead of real food…because the junk food is cheaper.

Interviewee 2: I'm on [disability benefits] at the moment, so sometimes I find it's a bit hard…I have to be very conscious of…and trying to think of not just buying food but planning meals and planning food throughout the month that will last throughout the month…sometimes I worry about running out of food and, because I'm diabetic, I can't just skip meals…I find I have to be more conscious of what I eat and when I eat…I find that a bit troubling.

Interviewee 3: It's all controlled by how much money you make…how much you pay rent. Most places [food banks] say you can come once every two weeks – and they give you one shopping bag of groceries and that's supposed to last you two weeks. If you don't have the funds, how are you supposed to eat? So you have to go to the food kitchens…you have your days kept busy running back and forth to find places to eat…some days you might not make it there…so you don't eat properly.

Interviewee 3: A lot of my food comes from the food bank. They only give you certain types of foods that don't really help you with diabetes, more or less go against your diabetes—a lot of sugar, cookies, stuff like that.

Interviewee 4: It's mostly pastas, heavy in starch, pasta, rice, pasta sauce…and cereals, stuff that's got sugar in it. There's not an alternative they give you.

Interviewee 5: You don't have enough fruits – juice is no good. The fruits are better, but you have zero access. They have fruit roll ups, they have junk food which I don't eat, so I give back most of the stuff.

Interviewee 6: The fact that I have the necessary facilities to cook…I've got a stove…but I never use it because…the person before [was]…I found this little thing and it'd blow up on me…I mean it looks nice, it's kept clean and it's spotless inside, but so is a hand grenade before you pull the pin too…I bought my own microwave…I use everything microwave or I eat out of a can cold.

Interviewee 7: I have to get…artificial sweetener now, too, and that's expensive compared to other stuff…I get the diet soft drinks, and I get juices. But then there's a lot of sugar in the juices, so I try and mix it with water, and then I find that not a lot of grocery stores have just a specific section. I've noticed that some of them, like the drug stores…have a certain section. Some of the items that are in the diabetic aisles…they are expensive too, right?

Interviewee 9: It'd be easier for me if I could just buy regular food, I don't have to buy special stuff that's low sugar and stuff. If I want it, I'd have to pay more for it.

Interviewee 8: For the past 4, 5 years…all the eye problems and everything else…it made it difficult to do things. You can't manage. You can't see to cook…the finger gets numb because of the diabetes. The nerves and the fingers, you cannot hold things…and even when you're buying stuff…you're holding it, but you can't really tell whether it's…good or [if] it's not good…I just…use the canned goods.

Interviewee 2: I hate going [to] the grocery store and getting…loads and loads…of groceries. I'd much prefer to shop every two or three days, but that's not really practical in terms of getting enough food and…making sure it keeps fresh…it's hard when…I've got some physical problems and hauling it around…is…difficult.

**Theme 2 (social isolation).**

Most participants were single and described the impact of food and eating alone on their well-being. Socializing incurred more cost on travel and clothes (see Interviewee 5). Because lack of funds limited social interactions, many felt isolated and depressed (see Interviewee 6).

While some considered eating out a special treat, especially for those who felt lonely and isolated, the impact of limited finances and having diabetes further restricted social interactions. Feeling the need to justify their food and beverage choices and to eat and drink differently from their friends were apparent barriers (see Interviewee 9).

Community food initiatives such as community kitchens and gardens, volunteering at food distribution centres, connecting to a church group, and attending drop-in meal programs were all means of connecting with others and were considered a valuable resource that helped to ameliorate both food insecurity and social isolation (see Interviewees 10 and 11).

Interviewee 5: I've…been inverting myself more to a cocoon?…I've stopped socializing…don't feel like it…cost of going, even if it's just subway or gas, it's money. So…I finally went to [an event] last week, because…I really had the feeling that if I didn't show up this time, they weren't going to invite me again…but people don't know…yes, I'm in between, going through a hard time, but that's about it. You can't admit that you're doing what you're doing. And most people don't recognize me dressed this way…I'm really careful where I go. So I basically don't go out unless I have to.

Interviewee 6: Since I can't always get what I really want, it adds to the depression…I can't go to the restaurant with my friends, “cause the guys are, like, “C”mon let me just take you for a cup of coffee or something outside”…I don't have the money to do that. I feel bad, I can't offer. A lot of times you have to go off your diet because, one, you can't afford it or, two, you have your meds. Thank God it's [diabetes medication] been covered, to compensate for what you don't have. I might go…to get a taco…or something that I haven't had in a long while, or go into the local grocery store. They have good pizza…and other things are cheap…it's also a treat…I've got a can opener that works, but how often…[do] I go…I've been eating alone in [my] apartment.

Interviewee 9: I really can't look forward to going out to eat…I'm having lunch with a friend of mine…I couldn't do that on my own…but he's treating me…I'd like to go out more, and I really can't, you know? I'm tired of saying “Well, we can't really go out…can we do this instead?” which doesn't cost any money…I feel a little bit guilty about that. …I don't know what people think of me…I can't really say “Oh I'm diabetic”, and I can't do this, this, and this, without making myself look like…I'm really, really sick…and I'm not really that sick, it just means that I've got to limit myself…[if] somebody says “Let's go to a club or somewhere”…I can't drink because you know when you drink…even beer, it's got sugar in it. If I have one beer, it's going to affect me the next day…if I go to a club and order a Diet Coke, you know they're just going to say “What's wrong with him?” I'm not going to really want to get into a big explanation about how sugar affects me.

Interviewee 10: I think mostly it's the social factor of eating alone. I don't like eating alone. So…there's a community dinner that I go to, my [church group]…I've gone there for years…I go to drop-ins with my friends for two reasons: one, because I like to eat with other people, and two, because I can't afford to buy food anymore…some of the places have really good food. Some of them have food that's very high in carbohydrates, which isn't good for diabetics…plus it puts weight on you.

Interviewee 11: I go to a church and they are helpful too…if I go to church on Sunday, somebody will take me home. Most Sundays I go home with somebody…that is the next supportive group, my church…they take me home and I'm fed for the day.

**Theme 3 (enhancing agency and improving resilience).**

Some participants considered their food insecurity to be temporary. One or more major life events, such as financial loss, job loss, marital separation, or divorce coincided with the onset of food insecurity. Many described survival strategies that buffered current adversity. For example, participants used positive self-talk, such as “Believe in your heart that it will be alright,” “My life will get back on track”, “You just live one day at a time”, and “I will survive”. Others expressed optimism through such phrases as “It's gotta be over…one way or the other”.

The role of healthcare providers is pivotal in enhancing agency and resilience. Participants drew on the heartfelt help and practical diabetes management strategies received from healthcare providers who they found to be “genuinely caring,” “gentle,” and “loving” (see Interviewee 14). Feeling cared for was referred to as “the human factor” and resonated in the examples participants gave, serving as empowerment to better manage their diabetes. Other participants appreciated when their healthcare providers inquired about issues not related to diabetes (see Interviewee 15).

Participants offered suggestions to care providers on what was helpful to them: “Keep information simple for people to understand”, “Listen to patients,” “Be positive, and encouraging and supportive”, and “Do not be judgmental”. Practical advice and counselling helped them regain their agency in managing diabetes and coping with food insecurity. More specifically, participants suggested “specific inexpensive menus,” “more lists, more recipes, and low-cost ideas of meals,” creative ways to share food, and alternatives to food choices (see Interviewee 9). In these ways, healthcare professionals can tailor their advice specifically to the needs of food insecure clients.

Participants demonstrated resilience by implementing various strategies to better manage their diabetes, given their circumstances. For example, several participants spoke about including protein with meals, saving time and costs by batch cooking and buying starchy foods in bulk, comparison-shopping for sale items, and buying nonbrand items from discount grocery stores. Participants acknowledged that taking their medications regularly helped to compensate for their limited control over food intake and compromised food choices (see Interviewee 6 and Interviewee 2).

Interviewee 14: I know from what I've seen with you and your staff, you people do genuinely care about us. That's a big step…it means a lot. I mean…since I was here last week till now, I've felt better about the whole situation because I know there [are] people out there that do actually care that I'm a diabetic…it really does make you feel better. It makes you wanna stay on track even more…And the way I see it, this is only human nature. Because everyone wants to feel loved somewhere, somehow, and when you know you have a group like that behind you, it, it makes you stronger.

Interviewee 15: Every time I go see [my family doctor]…he's not just like, “Yeah, okay you're here for the visit. Thank you very much. See you next month”…He'll ask me…“Emotionally, how do you feel? Is anything bothering you? Are you getting the shakes? Are you taking too much salt in?” So…I listen to him…he's good.

Interviewee 9: I'd say the most help I get is through this dietitian. You know, she'll tell me…“why don't you try this or you know, mix that with that?”…and, you know, I tell her my budget is limited and she'll say, “okay, this is cheaper, try that”, and it'd be things I would never think of.

Interviewee 6: Where the food is cheap…if you take your medication, it will counteract the stuff that you're not supposed to have…you eat what you can get, when you can get it. A lot of times you have to go off your diet because, one, you can't afford it or, two, you have your meds. Thank God [they're] covered, to compensate for what you don't have.

Interviewee 2: I think just keeping a positive outlook and just…making sure I take my medications…I guess I figure that the medication will make up for some of the lapses that I've done, because I know that some people are able to perfectly manage diabetes.

## 4. Discussion

Individuals living with diabetes who are food insecure face many challenges that greatly impact their ability to self-care [10–16]. Our findings demonstrate that these individuals have a limited ability to acquire, select, and prepare appropriate foods, in addition to maintaining consistent carbohydrate intake and meal spacing throughout the day. Barriers to observing an appropriate diet for diabetes management include financial constraints, a knowledge deficit for healthy meal planning on a limited budget, housing environments unconducive to food preparation and storage, inadequate community resources, and physical disabilities associated with comorbidities. The literature in Canada [10, 23] and Australia [7] confirms that many have insufficient income left after paying rent to purchase appropriate food. This was further compounded by the misperception that a “proper” diabetes diet requires “diet” foods that are low in sugar, contain artificial sweeteners, and are labelled “diabetic” [7]. Given the rising cost of healthy food [7], the circumstances of those who are food insecure can only get worse. Many study participants relied heavily on canned and convenient, high sodium, high carbohydrate foods. They stressed the inadequacy of food banks and community kitchens in meeting their special diet needs, a finding consistent with Tarasuk's research on community-based responses to food insecurity [23, 24]. López and Seligman suggest that the reliance on low-cost, energy dense foods and the inability to afford nutritious food can have a cascading effect not only on glycemic control, but also on depression, distress, and fatigue, all of which can negatively impact self-management behaviours [13]. Clinicians should therefore focus their recommendations on reducing portion size of foods that are available and accessible to them rather than focusing on food and beverage substitutions that may not be attainable [13].

Individuals with diabetes are more likely to have comorbidities than those without diabetes; it is estimated that all individuals with diabetes in Canada have some form of diabetic retinopathy, a frequent cause of legal blindness, and 40 to 50% of Canadians with type 1 or type 2 diabetes will manifest painful neuropathy within 10 years of diagnosis [4]. Several of our participants reported these complications and consequently had difficulty traveling to grocery stores, selecting fresh produce, and cooking nonprocessed foods due to sight and mobility impairments, creating a food insecure state in and of itself. Clinicians should therefore keep an inventory of meal or food delivery resources such as “Meals on Wheels,” “Grocery Gateway,” or “Heart to Home Meals,” all of which can assist an individual with physical disabilities to access, budget for, and prepare healthy foods without leaving the home and this has been supported by other research [13].

Based on our findings, we recommend that clinicians systematically screen for food insecurity, refer to the registered dietitian as needed, tailor the care plan, and identify increased health risks, particularly for hypoglycemia that often results from missed meals or inadequate carbohydrate intake. Treatment regimens should incorporate medications that have a lower risk of hypoglycemia and that can be adapted to unpredictable or inadequate food intake as a result of food insecurity [13, 15].

Trying to manage diabetes in conjunction with a low income led to social isolation for many; most of them lived in single-person households, a common characteristic of food insecure individuals [23]. Our participants had no one to share and prepare meals with; they also limited social interactions to save costs, increasing their risk of depressive symptoms. They used resources such as community kitchens, church groups, and food coops to cope with their food insecurity and social isolation. Canadian research has shown decreased psychosocial distress and increased food security among community kitchen participants [18]. We also recommend that clinicians incorporate more group-based learning opportunities such as workshops that focus on food budgeting, low-cost meals, and, as other authors have suggested [13], strategies for more affordable healthier substitutions (e.g., buying frozen instead of fresh vegetables). Including a hands-on component such as a food skills demo is essential in engaging and motivating clients to try these strategies on their own.

Research suggests that food insecurity is cyclical, shifting between periods of food scarcity and food adequacy [14] and that food insecurity can be either chronic or temporary [25]. Similar to research among low-income Canadians, most participants in our study viewed their food insecurity as a temporary setback [10] and employed various survival strategies to endure this setback. Participants also valued the “genuine” care and “support” received from health care providers. Valued relationships with healthcare providers can be the key for patients to regain their agency to manage their diabetes during bouts of food insecurity. Norwegian research suggests that healthcare providers can encourage diabetes self-management by employing an empathetic, individualized approach [26]. It also has been suggested to move beyond the “patient-centered approach” to one that is “empowering and partnering” and that builds an emotional relationship between the healthcare professional and the client [27]. Furthermore, a recent study reported that food insecure clients respond well to diabetes self-management support programs as evidenced by a decrease in hemoglobin A1c and an increase in self-efficacy and fruit intake [28].

The study has the following limitations. While we acknowledge that the experience between those with type 1 and type 2 diabetes differs, the goal of our paper was not to compare but to obtain the general challenges that these people experience. Also, there currently is a lack of research available to distinguish the major differences in experience between the two groups. We interviewed participants in a large urban centre and, therefore, our findings may not be transferable to people in small towns and rural areas. Future research should examine the benefits of physician screening for food insecurity and diabetes self-management programs tailored to food insecure clients as this has the potential to save significant medical costs and influence the future of diabetes care.

## 5. Conclusion

Food insecurity presents a great challenge to diabetes self-management, an already complex chronic illness. In our study, participants faced multiple barriers to accessing appropriate foods: insufficient income, misperceptions about healthy food choices, multiple comorbidities, and inadequate cooking facilities that cumulatively impact food acquisition, selection, and preparation. Social isolation compounded these barriers, although it was somewhat buffered by the coping strategies they used and by the community food initiatives and social support networks they were able to access. Without access to healthy food, the identified barriers can potentially result in fatigue, decreased social well-being, and increased health problems, ultimately discouraging an individual to practice self-care behaviours (e.g., blood glucose monitoring, physical activity, and healthy coping). Healthcare providers should be aware of the challenges that food insecurity poses for people with diabetes, as well as the potential they have to optimize their encounters with patients. Our findings underpin the importance of understanding diabetes through the perspectives of patients' lives and tailoring diabetes management plans and community programs within the context of food insecurity.

## Figures and Tables

**Table 1:** Sociodemographic characteristics of participants.

	Number (*n* = 21)
Gender	
Women	10
Men	11
Ethnicity	
Caucasian	12
Caribbean	5
African	2
Middle Eastern	2
Place of birth	
Canada	12
Outside of Canada	9
Age	
20–30	1
31–40	1
41–50	7
51–60	9
61–70	3
Marital status	
Married/common-law	2
Single	9
Divorced/separated/widowed	10
Education	
High school or less	7
University or less	4
College or less	9
Graduate school	1
Duration of diabetes	
≤5 years	11
6–10 years	4
11–20 years	2
21–30 years	2
>30 years	2
Type of diabetes	
Type 1	1
Type 2	20
